# The Global Evidence Mapping Initiative: Scoping research in broad topic areas

**DOI:** 10.1186/1471-2288-11-92

**Published:** 2011-06-17

**Authors:** Peter Bragge, Ornella Clavisi, Tari Turner, Emma Tavender, Alex Collie, Russell L Gruen

**Affiliations:** 1 Department of Surgery, Monash University, Level 6, 99 Commercial Road, Melbourne, Victoria, 3004, Australia; 2 National Trauma Research Institute, Level 4, 89 Commercial Road, Melbourne, Victoria, 3004, Australia; 3 Australasian Cochrane Centre, Monash University, Level 6, 99 Commercial Road, Melbourne, Victoria, 3004, Australia; 4 Institute for Safety, Compensation and Recovery Research, Level 6, 499 St Kilda Road, Melbourne, Victoria, 3004, Australia

## Abstract

**Background:**

Evidence mapping describes the quantity, design and characteristics of research in broad topic areas, in contrast to systematic reviews, which usually address narrowly-focused research questions. The breadth of evidence mapping helps to identify evidence gaps, and may guide future research efforts. The Global Evidence Mapping (GEM) Initiative was established in 2007 to create evidence maps providing an overview of existing research in Traumatic Brain Injury (TBI) and Spinal Cord Injury (SCI).

**Methods:**

The GEM evidence mapping method involved three core tasks:

1. Setting the boundaries and context of the map: Definitions for the fields of TBI and SCI were clarified, the prehospital, acute inhospital and rehabilitation phases of care were delineated and relevant stakeholders (patients, carers, clinicians, researchers and policymakers) who could contribute to the mapping were identified. Researchable clinical questions were developed through consultation with key stakeholders and a broad literature search.

2. Searching for and selection of relevant studies: Evidence search and selection involved development of specific search strategies, development of inclusion and exclusion criteria, searching of relevant databases and independent screening and selection by two researchers.

3. Reporting on yield and study characteristics: Data extraction was performed at two levels - 'interventions and study design' and 'detailed study characteristics'. The evidence map and commentary reflected the depth of data extraction.

**Results:**

One hundred and twenty-nine researchable clinical questions in TBI and SCI were identified. These questions were then prioritised into high (n = 60) and low (n = 69) importance by the stakeholders involved in question development. Since 2007, 58 263 abstracts have been screened, 3 731 full text articles have been reviewed and 1 644 relevant neurotrauma publications have been mapped, covering fifty-three high priority questions.

**Conclusions:**

GEM Initiative evidence maps have a broad range of potential end-users including funding agencies, researchers and clinicians. Evidence mapping is at least as resource-intensive as systematic reviewing. The GEM Initiative has made advancements in evidence mapping, most notably in the area of question development and prioritisation. Evidence mapping complements other review methods for describing existing research, informing future research efforts, and addressing evidence gaps.

## Background

All stakeholders in health care like to have research evidence to inform their decision-making. Some decisions relate to questions that are narrowly focused, such as the effectiveness of an intervention or the accuracy of a diagnostic test. Others are much broader, such as the relative effectiveness of various treatment options available for a particular condition. The methods of systematic reviews are well developed for the former group, but the latter poses many challenges for locating, organising, collating and synthesising information into a useful, understandable format, as it requires an organised up-to-date reference source of research evidence addressing broad content areas.

For this task, existing evidence resources seem generally inadequate. Finding all relevant publications on a topic using PubMed-type databases is a long and laborious task. Systematic reviews are rarely completely up-to-date. Being the product of a largely investigator-driven collaboration, the Cochrane Library doesn't necessarily contain reviews encompassing all questions in a given topic area. Furthermore neither searches for primary studies, nor systematic reviews are well suited to identifying important evidence gaps.

Techniques for describing existing evidence in a broad content area are relatively new, and have been called evidence mapping and scoping studies [1,2]. How they compare with systematic reviews is shown in Table 1. Importantly, evidence mapping and scoping does not extend to the quality appraisal and synthesis techniques associated with systematic reviews. Scoping is further distinguished from mapping by the inclusion of research results in the description of relevant evidence. Three publications have outlined methods for mapping or scoping a content area [3-5], which have included three core tasks:

1. Setting the boundaries and context of the topic area in question;

2. Searching for and selection of relevant studies; and

3. Reporting on yield and study characteristics.

**Table 1:** Methods of identifying and collating research evidence

Method	Definition	Purpose	Breadth	Depth of process
Systematic Review	"an overview of primary studies which contains an explicit statement of objectives, materials and methods and has been conducted according to explicit and reproducible methodology." [22]	Summarise overall quality and results of a body of research; inform clinical practice	Addresses a focused clinical question [13]	In depth searching, quality appraisal and synthesis of studies relevant to the identified clinical question

Scoping Study	Overview of "the key concepts underpinning a research area and the main sources and types of evidence available" Mays et al. 2001; cited in [3]	Examine the extent, range and nature of research activity; identify research gaps [3]	Covers a broad topic area	Identifying boundaries and context of the area under study, followed by searching, collation and summary of study characteristics and results with no quality appraisal or synthesis

Evidence Mapping	The systematic organisation and illustration of a broad field of research evidence [3,5]	Characterise the breadth, depth, methodology of relevant evidence and make this readily accessible [5]; identify research gaps	Covers a broad topic area	Identifying boundaries and context of the area under study and providing a description of yield, interventions, study design and study characteristics

In 2007 the Global Evidence Mapping (GEM) Initiative was established with funding from the Victorian Neurotrauma Initiative (VNI), a research funding agency established by the Victorian Government's Transport Accident Commission and Department of Innovation Industry and Regional Development [6]. The GEM Initiative developed as a collaboration of clinical, research and policy stakeholders to provide an overview of existing research about Traumatic Brain Injury (TBI) and Spinal Cord Injury (SCI).

The GEM Initiative aimed to map the available research for the prehospital, acute inhospital and rehabilitation phases of care for TBI and SCI. The breadth of clinical research questions and volume of studies in these areas is considerable. The evidence mapping approach was thought to be the most appropriate method because its main purpose is to provide an overview of a broad range of research questions and identify evidence gaps [3-5]. In creating the SCI and TBI evidence maps, the GEM Initiative drew on and advanced evidence mapping methods. Importantly, we developed an in-depth question development process, which enabled us to satisfy a key project aim - the identification of evidence gaps - by comparing and contrasting stakeholder-driven clinical research questions with published literature.

This article focuses on the GEM Initiative's evidence mapping methods and illustrates how these methods were applied to neurotrauma (TBI and SCI) topics. The results of the mapping process itself are also briefly described.

## Methods

**Figure 1:**
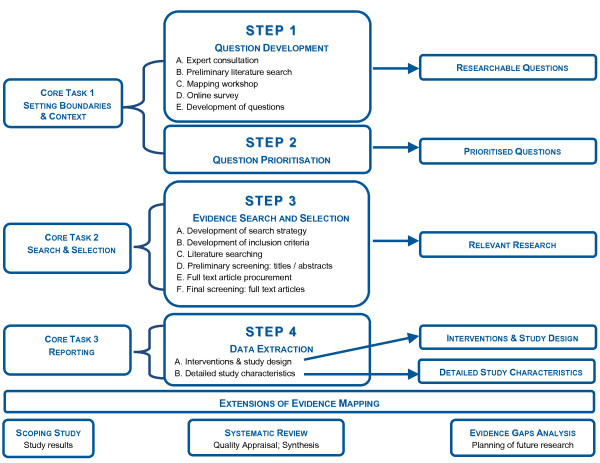
**GEM Initiative Evidence Mapping Methods**.

Figure 1 summarises the steps of the GEM Initiative evidence mapping process (centre of figure), their relationship to previously published core evidence mapping tasks (left of figure) and the resulting outputs (right of figure). The figure also illustrates the boundary between evidence mapping and the other secondary research methods described in Table 1.

Because evidence mapping outputs have stand-alone merit and application, the GEM evidence mapping process, like previously published evidence mapping methods, is not an 'all or nothing' proposition; the number of steps undertaken and the breadth of the topic area covered can therefore vary according to available resources. An evidence map can form the basis of a scoping review or number of individual systematic reviews, as evidence mapping produces source materials for both these activities (Table 1). Further, analysis of the evidence gaps identified in the mapping process can aid planning of future research by reducing duplication of research effort within topic areas, and focussing research efforts on high priority topics (Figure 1).

Following is an in-depth description of the GEM Initiative evidence mapping methods, illustrated with specific examples from our evidence maps. Emphasis has been placed upon the unique aspects of these methods; the use of a mapping workshop to identify clinical research questions, development of questions, question prioritisation and the two levels of data extraction and presentation.

### Core task 1: Setting the boundaries and context of the map

The GEM Initiative approached this task using two distinct steps - question development and question prioritisation.

#### STEP 1: Question Development

The question development phase involved four broad data collection exercises designed to gain an overview of the scope of the field and gather perceptions of key stakeholders - patients, carers, clinicians, researchers and policymakers - regarding the key clinical issues in the field of interest. The data gathered from these exercises was used to develop searchable clinical questions.

##### 1A: Expert Consultation

Consultation with experts has been identified as a useful strategy for setting the boundaries and context of an evidence map [4,5]. Experts identify specific terminology, which aids in search strategy development, and outline who has stakeholder interests. This information informs subsequent stages of the mapping process [4,5].

To frame the TBI and SCI evidence maps, the GEM Initiative consulted with local and international clinical and research leaders in these fields. These consultations involved introducing the GEM Initiative and the concept of evidence mapping, followed by an unstructured discussion designed to enhance our understanding of specific aspects of TBI and SCI care. For example, our discussion with experts in SCI rehabilitation encompassed common problems experienced by SCI patients in this phase of care and the healthcare disciplines involved in managing these.

Although the GEM Initiative focused on clinicians and researchers, the other key stakeholders with expert knowledge in this area - patients, carers and policymakers (who were involved in later stages in the GEM process) - can add different perspectives to this process and could also be involved at this stage.

##### 1B: Preliminary literature search

To identify existing relevant reviews and guidelines relating to TBI and SCI in each of the phases of care, a preliminary literature search was conducted by a GEM Initiative project officer who had extensive Cochrane systematic review experience. Major medical databases were searched using relevant Medical Index Subject Headings (MeSH) and other terms based upon the clinical knowledge of the GEM researchers. The focus of the search was on reviews and guidelines rather than primary studies. Relevant reviews and guidelines were used to identify clinical questions, describe the extent to which they had been addressed by existing literature and identify additional key literature search terms. This information further developed the scope of the map.

##### 1C: Mapping Workshop

To ensure the evidence maps were comprehensive and useful, key stakeholders involved in all phases of TBI and SCI patient care - patients, carers, clinicians, researchers and policymakers - were invited to participate in mapping workshops. This is an enhancement on the techniques for obtaining expert opinion described by Katz et al. [5].

The primary aim of the mapping workshops was to gather stakeholder perspectives on key clinical questions, outcomes, patient factors and contextual factors affecting TBI and SCI care. A total of four mapping workshops were conducted; one each for the pre-hospital and rehabilitation phase of care in which both SCI and TBI stakeholders were involved; and separate TBI and SCI workshops dealing with the acute inhospital phase of care.

The nominal group technique [7] was used. This involved individual, anonymous generation of ideas followed by group discussion. The nominal group technique overcomes the inhibiting influences that can operate when group interaction is used to generate ideas [7]; examples of such inhibiting influences include a health professional deferring to a senior colleague rather than offering their own opinion, or a patient not wanting to highlight issues that could be construed as criticism of their clinicians.

A professional facilitator with experience in the nominal group technique ran the workshops. The mapping workshops varied in length from one to two hours according to the breadth of the field and participant availability. A consistent workshop format was employed comprising:

• *Introduction: *Aims, key definitions, confidentiality/consent procedures.

• *Brainstorming: *Writing of ideas on individual adhesive notes, without discussion. The PICO format ('Problem', 'Intervention', 'Comparator', 'Outcome') was suggested but not mandatory. Clinical issues could include diagnosis, prognosis, interventions, and service delivery/organisation. The adhesive notes were then placed on large sheets around the room.

• *Prioritisation: *During a short refreshment break, GEM Initiative researchers grouped similar notes. Participants then prioritised the notes by placing a limited number of adhesive dots next to the notes they perceived as most important.

• *Discussion: *Open discussion was conducted on the ideas generated, the results of the prioritisation and any further issues identified. This was a valuable way of gaining insight into other perspectives and elaborating, modifying and developing consensus [7]. Where time permitted, the issue of contextual factors (for example rural or urban location, socioeconomic status) and their influence on the feasibility of delivering an intervention, the intervention itself, outcomes, or other aspects of care was discussed.

• *Conclusion: *Participants were thanked and invited to participate in subsequent question development and prioritisation activities and/or receive a copy of the final report.

The mapping workshops resulted in an unstructured collection of ideas and PICO terms of interest as well as a sense of the relative importance of these ideas.

##### 1D: Online survey

An online survey was used to augment the information gathered in the mapping workshops. Only one published evidence mapping method has incorporated the use of a survey. The GEM Initiative evidence mapping survey built on the method described by Katz et al. [5] by placing an emphasis on the PICO elements that form the basis of searchable clinical questions and, like the mapping workshop, bringing patients into the process of generating ideas and questions.

Survey recipients were identified through GEM clinical and research contacts, primary searching of hospital and academic websites and organisational mailing lists, including a mailing list of clinicians and researchers on the VNI database to which GEM staff were blinded. The survey comprised three sections:

1. *Introduction and background: *Brief background to the project, purpose, confidentiality and contact information.

2. *Survey of PICO terms of interest: *Participants were asked to fill in a minimum of two blank PICO templates identifying a series of clinical problems, therapies and/or outcomes that they considered important in their specialty area.

3. *Demographic details: *Profession/patient group, years experience or years a patient, extent of research/clinical involvement in TBI/SCI, geographical location, gender.

The survey data were more closely aligned to the PICO format than the mapping workshop data, as the survey responses were mandated under PICO headings.

##### 1E: Development of questions

These four activities (1A -1D) generated a very large volume of unstructured ideas including single words, PICO fragments, statements and questions. They varied in format, language, perspective and emphasis because a range of stakeholders was involved in the process.

Organisation of this heterogeneous data was required in order to promulgate a key aim of the GEM Initiative - the identification of evidence gaps - by searching for literature pertaining to the identified issues. We addressed this need by transforming the data into a list of 'answerable' clinical research questions. An 'answerable' question seeks specific knowledge, is framed to facilitate literature searching and therefore, follows a semi-standardised structure [8,9]. The challenge of representing broad stakeholder perspectives in answerable question form has been recognised by Bates et al. [4]. The GEM Initiative used an in-depth, systematic process for transforming the data from steps 1A - 1D into answerable clinical research questions that involved:

1. Entering data into an electronic database (Microsoft Excel).

2. Coding the clinical problems described using codes from the International Classification of Functioning, Disability and Health (ICF) [10,11]. This comprehensive framework was suitable for identifying the broad range of clinical issues across multiple phases of TBI and SCI care; for other evidence mapping topic areas, other taxonomies may be more appropriate for coding purposes.

3. Collating coding information to identify the range and frequency of all codes represented in the data.

4. Conducting a series of half-day question construction meetings in which the GEM Initiative research team transformed the data into a list of answerable clinical research questions encompassing all ICF codes represented in the data.

The example in Table 2 illustrates the transformation of data from steps 1A - 1D into an answerable clinical research question using this approach.

**Table 2:** Transformation of question development data into an answerable clinical research question

Data source	Example of data	**ICF code **[10,11]	Resulting answerable clinical research question
1A: Expert consultation	"Spasticity"	b735: Muscle tone functions	
	
1B: Preliminary literature search	"What are the best pharmacological treatments to manage TBI spasticity?" "How effective is intrathecal baclofen, botulinum toxin type A, passive exercises etc?"	b735: Muscle tone functions	**What are effective interventions for preventing and managing skeletal muscle spasticity?**
	
1C: Mapping workshop	"Problem: spasticity management" "Decreased joint range of motion and integrity" "Spasticity causing functioning or safety problem"	b735: Muscle tone functions	
	
1D: Online survey	"P: Spasticity I: Pharmacological management of tonal changes, surgical intervention, positioning techniques, gentle movement program, patient/family education O: Maximise passive and/or active joint range and maintain optimal joint integrity"	b735: Muscle tone functions	

ICF: International Classification of Functioning, Disability and Health

A range of question development strategies have been reported in published evidence mapping projects. Arksey [12] used qualitative analysis to identify the key topics and issues arising from stakeholder consultation in her scoping review of interventions for carers of people with mental health problems. Bates et al. [4] used a PICO-style approach to frame inclusion and exclusion criteria for literature search and other mapping tasks. Similarly, Arksey et al. [3] discussed the importance of defining key question parameters and recommended framing such parameters to maximise breadth of literature coverage.

The GEM Initiative approach to question development built upon these strategies by using the ICF framework to organise large amounts of unstructured data and detailing the specific tasks involved in transforming this data into answerable clinical research questions. This approach facilitated the development of a large number (129) of answerable research questions across an entire research field. The comprehensive question development approach used by the GEM Initiative ensured that all of the ideas identified by the key stakeholders in steps 1A - 1D - patients, carers, clinicians, researchers and policymakers - were represented in the final list of answerable clinical research questions for each phase.

#### STEP 2: Question Prioritisation

Bates et al. [4] recognised that the evidence mapping process involves calibrating the answerable questions developed against the resources available to map these questions. Given the number of questions identified in the GEM Initiative question development process, there was a clear need to prioritise these as our project resources did not enable all steps of the mapping process to be applied to all identified questions.

The GEM Initiative involved the key stakeholders from the question development process in prioritising the list of clinical research questions from Step 1. An online survey of patients, clinicians and researchers was conducted. Survey recipients included participants from the mapping workshop, the GEM advisory group and other representatives with expert knowledge of TBI and SCI. Survey respondents were asked to rank each searchable clinical question on three domains - clinical importance ('How clinically important is this question?'), novelty ('Does this question represent an emerging area of clinical practice?') and controversy ('What is the level of variability regarding opinion and/or practice for this question?') - using a scale of 1 to 4, where:

**1 **= not at all

**2 **= a little

**3 **= a moderate amount

**4 **= high.

The questions were then ranked into two broad categories:

• ***High priority questions: ***Questions where the majority of respondents ranked 'Clinical Importance' as high **AND **('Novelty' **OR **'Controversy' as high or moderate).

• ***Low priority questions: ***All other questions.

This ranking procedure was arbitrary and driven by the imperatives of the GEM Initiative project; other research groups may use other ranking parameters and divisions consistent with the focus of their research activity. For example, cost-effectiveness or feasibility of undertaking primary or secondary research, rather than clinical importance, may be deemed the most important determinants of which questions to pursue.

### Core task 2: Searching for and selection of relevant studies

#### STEP 3: Evidence Search and Selection

The evidence search and selection methods used by the GEM Initiative were consistent with accepted principles of systematic reviewing. Specifically, these were:

1. In-depth search strategies using applicable MeSH headings and keywords [9,13].

2. Pre-determined inclusion criteria [4,13].

3. Searching a broad range of medical databases.

4. Review of retrieved citations/full-text articles against the question-specific inclusion criteria by two independent reviewers to reduce bias [3,13].

As such methods are extensively documented in systematic review methodology literature, only a brief description of their specific application to the GEM Initiative follows. Examples are contained in Appendix 1.

##### 3A: Development of search strategy

An information specialist was used to develop and run searches and collate search results. Search strategies were built by combining at least two of:

• Core terms representing TBI and SCI

• Filter terms, for example to target rehabilitation literature

• Intervention terms specific to the clinical questions being mapped.

GEM Initiative researchers with a clinical background (the research team included two medical doctors, a physiotherapist and a nurse) contributed their clinical knowledge of the subject areas to search strategy development. Abridged versions of the search strategy were used for the databases that had a simpler search interface or smaller content, such as non-English databases, as these databases could not accommodate a large number of search terms [4,14].

##### 3B: Development of inclusion criteria

*A priori *eligibility criteria were developed regarding study type (systematic reviews and a broad range of primary study designs encompassing therapy, diagnosis and prognosis were included; animal, laboratory, cadaver and simulation studies were excluded), participants (explicit definitions of TBI and SCI) and phase of care (prehospital, acute inhospital, rehabilitation; injury prevention research was excluded).

Given the breadth of topics covered, question-specific refinements to the inclusion criteria were sometimes required after preliminary database searching and review; for example, to determine which clinical sub-categories of a particular disorder were most relevant. Such post hoc refinements, which can only occur when some literature has been examined [3], also aided in the classification of eligible studies. For example, the management of spasticity in TBI could be divided into surgical, pharmacological and physical therapies. Where necessary, changes to the search strategy were made to reflect these alterations and refinements. A similar iterative approach has been adopted by others [4].

##### 3C: Literature searching

Major medical databases (including non-English) and trials registries were searched for all phases of care covered by the GEM Initiative. Searching of further databases tailored to the specific needs of each phase/question was also conducted. For example, for the question pertaining to spasticity, three additional databases were searched to reflect the multidisciplinary nature of this intervention in addition to the databases routinely searched (Appendix 1).

For all searches there was no restriction on year of publication or language. For each question all of the chosen databases were searched on the same day and a reference library (Endnote) of search results by database was compiled. The reference library was then de-duplicated for the purpose of screening and selection.

##### 3D: Preliminary screening: titles/abstracts

Retrieved citations were reviewed against the question-specific inclusion criteria by two independent reviewers. Where necessary, a third reviewer was used to resolve disagreements by breaking a tie.

##### 3E: Full-text article procurement

Full-text articles identified during screening were procured either directly or via order from university/hospital libraries.

##### 3F: Final screening: full-text articles

Full-text articles were reviewed against inclusion criteria as described above (3D).

Records of screening and selection decisions (citations excluded; citations identified for full text review; citations agreed upon for full text review; studies excluded upon full-text review; studies identified for inclusion in evidence map; studies agreed upon for inclusion in evidence map) were systematically kept.

As a search of all the major and auxiliary databases may inadequately reflect the total relevant research on any topic [15,16], reference lists of relevant articles were systematically searched by GEM reviewers [13] and expert clinicians were consulted to identify further potentially relevant articles.

### Core task 3: Reporting on yield and study characteristics

The GEM Initiative undertook two levels of evidence mapping reporting; an overview of interventions (where relevant) and study design of included studies and a more in-depth exploration of study characteristics. In each case, the output tables and text identified and articulated evidence gaps by describing how the relevant literature addressed the research question. These levels of evidence mapping are described and illustrated below with examples from the GEM Initiative evidence maps.

#### STEP 4: Data Extraction

A relational data extraction database was created (Microsoft Access), which could be interrogated to explore and query the evidence map [4].

For the **prehospital **evidence map, an in-depth data extraction and review of study quality was undertaken. However this process proved to be unfeasible once the volume and breadth of literature in subsequent phases became apparent. Furthermore, it was felt that quality appraisal, summary of findings and specific outcomes of individual studies was better conducted within a formal systematic review, and we have distinguished evidence maps and systematic reviews in part on this level of data extraction and reporting as outlined earlier. This resulted in the development of the two data extraction protocols that were used in the **rehabilitation **and **acute inhospital **evidence maps:

##### 4A: Interventions and study design

Extracting to this level involved identifying the number of studies by study design for each intervention addressing the question. Where the question did not pertain to an intervention, relevant studies were listed by study design only. For all phases of the project, study design was classified according to the definitions outlined in the Australian National Health and Medical Research Council (NHMRC) levels of evidence [17] and Straus et al. [9]. A brief commentary on evidence summarised the key findings regarding the yield and breakdown of study interventions and designs (Table 3).

**Table 3:** Example of 'interventions and study design' output: Prevention and management of skeletal muscle spasticity in the rehabilitation phase of TBI

Intervention	n	SR	RCT	X-over	Cohort	ITS	Case series	Case report
Casting/splinting	16	1		2	1	2	5	5

TENS	2						1	1

Baclofen: Intrathecal long term	16		1			3	11	1

Baclofen: Intrathecal bolus/test dose	5		1			3		1

Baclofen: Oral	1						1	

Botulinum toxin (Botox)	10		3			1	2	4

Clonidine	1		1					

Lower leg casting/splinting & Botox	1		1					

Cryotherapy	1						1	

Phenol nerve block	1						1	

Weight-bearing gait retraining	1							1

Divolproex sodium	1							1

Bobath treatment	1							1

Voice and respiration treatment	1							1

Rhythmic, passive movement	1							1

Seating	1							1

Combination of therapies	1							1

SR: Systematic Review

RCT: Randomised Controlled Trial

X-over: Crossover trial

ITS: Interrupted Time Series

TENS: Transcutaneous Electrical Nerve Stimulation

Commentary on evidence

Over half of the 61 studies investigated pharmacological/neurotoxin therapies for managing spasticity in TBI. However, no systematic review of this literature was identified. The next most common intervention under investigation was casting/splinting. One systematic review of this intervention was identified [23]. Only ten of the 61 studies investigated therapies other than pharmacological/neurotoxin agents or casting/splinting.

##### 4B: Detailed study characteristics

The following data were extracted: study design, country, sample size, source population, interventions, outcome measures, patient factors (demographics, injury classification). This facilitated a more in-depth evidence map for the clinical question, as reflected by both the output table and the scope of the commentary on evidence (Table 4).

**Table 4:** Example of 'detailed study characteristics' output (extract only): Effective interventions for optimising bowel function in the rehabilitation phase of SCI

Reference	Study Design	Country	n	**Pop**.	Patient Group	Condition	Intervention	Outcomes
Ayas et al. 2006 [24]	Case series	Turkey	24	All SCI	Adult	Neurogenic bowel	Abdominal massage	Mean time for bowel evacuation; Frequency of defecation; Faecal incontinence; Abdominal distension; Abdominal pain; Difficult intestinal evacuation

Furusawa et al. 2007 [25]	Case series	Japan	15	All SCI	Adult	Autonomic Dysreflexia	Bowel program involving manual removal of stool	BP; Pulse; Symptoms of cervical SCI

Luther et al. 2005 [26]	Retrospective cohort	USA	370	All SCI	Adult	Neurogenic bowel	Bowel care program; Colostomy	Training for bowel care program; Quality of life; Subjective complication rates

Commentary on evidence

Two systematic reviews were identified in this topic area covering colostomy and ileostomy and multimodal bowel management and over half (21) of the eligible primary studies were case series. Five studies recruited over 100 patients. Four of these were exclusively SCI populations, however, the largest study in the evidence map that was on multimodal bowel management (n = 837), comprised a mixed population with no subgroup analysis. The majority of studies assessed specific physiological parameters, such as time to flatus and number of bowel evacuations. Only seven studies assessed quality of life measures.

(Note: this commentary refers to studies not contained in the above table, which is an extract of a table containing the 34 included studies).

Use of these two data extraction methods facilitated a broad coverage of clinical questions in these areas within project resources. The decision regarding which data extraction method to use involved consideration of a range of factors including the nature and complexity of the question and the need to balance consistency, comprehensiveness and question coverage across both TBI and SCI topics.

Complete versions of tables and reports exemplifying both styles of data extraction are available at http://www.evidencemap.org. This site contains a searchable database of all TBI and SCI topics for which an evidence map has been completed. For each topic, either the output tables ('maps') alone (as illustrated in Tables 3 and 4) can be downloaded, or a report is available containing the maps plus a background to the topic, the inclusion criteria for search, the search strategy and yield, a description of the map, a commentary on evidence and a reference list. The date of last update is also listed for each topic.

## Results

Although this paper focuses on the steps of the evidence mapping process, a brief overview of the findings of the GEM mapping process itself illustrates its usefulness in identifying evidence gaps.

### Question development

Since the inception of the GEM Initiative, a total of 129 answerable clinical research questions have been generated, covering the prehospital (n = 30; 17 TBI, 13 SCI), acute inhospital (n = 37; 18 TBI, 19 SCI) and rehabilitation (n = 62; 26 TBI, 36 SCI) phases of TBI and SCI care.

### Question Prioritisation

The prioritisation procedure undertaken in the GEM Initiative resulted in 60 of the 129 clinical research questions being identified as high priority; 30 in each of TBI and SCI.

### Evidence Search and Selection

Since 2007, 58 263 abstracts have been screened, 3 731 full text articles have been reviewed and 1 644 relevant neurotrauma publications have been identified.

### Data Extraction

Data extraction has been completed to produce evidence maps for fifty-three high priority questions. For 11 of the 53 questions (3 TBI, 8 SCI), gaps in primary research evidence were identified:

• For six questions (2 TBI, 4 SCI) no studies were identified.

• For a further four questions pertaining to interventions (1 TBI, 3 SCI), only case series, case study or cross-sectional study designs were identified. These represent neurotrauma research topics in which comparative studies (for example, Randomised Controlled Trial, Pseudo-Randomised Controlled Trial, Case-Control Study) are needed, as reflected by the NHMRC levels of evidence for intervention studies [17].

• For a further question pertaining to prognosis in SCI, no prospective cohort studies were identified, which are the highest ranked primary study for investigating prognosis [17].

TBI topics with primary evidence gaps included prehospital therapeutic hypothermia and participation in leisure and social activities. SCI topics with primary evidence gaps included prehospital antiemetic medications and interventions for sleep disordered breathing.

A further 12 questions (9 TBI, 3 SCI) contained secondary evidence gaps; more than one comparative study were identified, but no systematic review. These are neurotrauma research topics in which research synthesis may be warranted. TBI topics with secondary evidence gaps included prehospital hyperventilation and interventions for minimising drug and alcohol misuse. SCI topics with secondary evidence gaps included interventions for atelectasis/chest infections and interventions for optimising nutrition and body weight.

## Discussion

The GEM Initiative has produced evidence maps that may be useful to research funders, researchers, clinicians and other research consumers. An extensive list of prioritised research questions in a topic area, even in the absence of study retrieval and data extraction, is a potential springboard for research, research funding and policy development. Because the question development and prioritisation process is driven by consultation with key stakeholders - patients, carers, clinicians, researchers and policymakers - this list is more likely to be relevant to real-world issues than research questions driven solely by academic researchers. Our experience of the power of engaging with a wide stakeholder group is shared by Arksey et al. [3], who reflected on the additional insights that this approach gives to the process. Developing and prioritising research questions by consulting with a number of stakeholder groups carries the dual advantage of adding breadth to the research topics identified and diluting the potential influence of individual or stakeholder group biases on the question development process.

Funding agencies can use evidence maps to access completed/ongoing studies in a topic area; for example, studies using intrathecal baclofen for spasticity in TBI. This information can aid in evaluating the need for further funded research when reviewing a grant application. The collection of evidence maps across a whole topic area (for example, TBI rehabilitation) also highlights areas of high and low research activity, potentially informing strategic research funding decisions. The VNI exemplified this application of evidence maps through its explicit instructions to funding applicants to consult the GEM evidence maps in the process of preparing their applications [18]. Other research-informed agencies could also benefit from evidence maps - for example, a policy-maker could gain an understanding of the volume and nature of research in a topic area to aid in funding or other policy decisions.

Researchers can use evidence maps to inform research decisions and designs. Evidence maps parallel all steps of a traditional systematic review except in-depth quality appraisal and synthesis. Systematic reviewers can therefore use evidence maps as a resource for updating an existing systematic review, or to evaluate the feasibility and resource requirements of a systematic review based upon the search yield, and proceed to full study appraisal and synthesis using this resource. One example of such a review is a Cochrane review of interventions for managing skeletal muscle spasticity, which built upon the GEM evidence map of this topic [19]. As illustrated in the results, an evidence map of a specific topic can also avoid duplication of primary research and determine where further primary studies are needed or conversely, identify if a systematic review of existing studies may be more appropriate. Studies included in an evidence map could also aid primary research planning, for example by describing outcome measures or research protocols.

Clinicians can use evidence maps to rapidly access research information pertaining to specific populations and interventions. For example, a case manager could access literature about the effectiveness of community integration programs for TBI specifically in paediatric populations, or focusing on computer-based interventions. However, it should be emphasised that evidence mapping does not purport to guide clinical practice, as the studies in the GEM Initiative evidence maps have not been subject to quality appraisal or had their results synthesised.

This limitation of evidence mapping necessitates caution especially in cases where a large volume of evidence is identified for a topic. For example, for eleven of the GEM topics (4 TBI, 7 SCI) over 50 relevant English articles were identified. These represent neurotrauma research topics in which knowledge translation research, rather than more primary or secondary studies, may be appropriate. However, this cannot be surmised from the yield alone, but by combining this with an examination of the quality of the comparative studies and/or systematic reviews. Should the research be of high quality and consistency, knowledge translation research may be warranted. If this is not the case, further primary or secondary studies are required - just as in cases where there is a low volume of evidence. Avoiding the potential hazard of misreading evidence maps is best facilitated by transparency in the description of the purpose and methods of evidence mapping, in particular by drawing a clear distinction between evidence mapping and systematic reviewing (Figure 1); ensuring that the results of the evidence maps themselves are not presented as definitive statements on the direction of research evidence; and promoting research that fills the evidence gaps identified by the mapping process.

Identification of and prolonged engagement with the likely end-users (clinicians, researchers, funding agencies) starting early in an evidence mapping project enables tailoring of evidence mapping activities and outputs to their priorities. Although there was provision in the evidence mapping process for the identification and mapping of non-intervention questions, the existing GEM evidence resource predominantly contains maps pertaining to interventions. This could be in part due to the use of PICO to guide question development; it may be a reflection of the priorities of those involved in question development; or it could be a simple illustration that most clinical research questions are intervention-focused. In this context, some tailoring to the evidence mapping process could be necessitated (for example, less emphasis on PICO) if outputs focused on non-intervention issues are identified as important during initial consultation with end-users.

There are many resource-intensive steps in evidence mapping. As described, the GEM Initiative evidence maps involved the generation of 129 clinical questions and independent review of tens of thousands of abstracts and thousands of full-text articles by the 3.5 EFT project staff. A similar level of staffing has been reported by Arksey et al. [3]. Preliminary calculations performed by the GEM Initiative, based on time spent to develop detailed search strategies, search databases and select studies, indicate a resource cost of 1.5 minutes for each citation reviewed [20], plus the costs of setting the boundaries and context of the map and reporting on yield and study characteristics.

Our experiences in this project combined with the available cost data reinforce the statement by Arksey et al. [3] that "the scoping study should not be seen as a cheap alternative to the systematic review" (p. 29). Primarily this is because any savings in resource use from not synthesising and appraising studies to the depth of a systematic review are easily overtaken by the costs associated with mapping a much broader topic area. Given the resource requirements of evidence mapping, efficiencies (that do not compromise scientific rigor) must be explored in order to ensure the long-term sustainability of evidence mapping.

## Conclusions

Since its inception in August 2007, the GEM Initiative has advanced the field of evidence mapping. The in-depth question development and prioritisation methods - in particular the use of mapping workshops to engage a broad range of key stakeholders - result in evidence maps that are more likely to be relevant to patients, carers, clinicians, researchers and policymakers. This question development method could easily adapt to any situation requiring the generation of clinical questions.

The study search and selection methods parallel accepted systematic review approaches, creating potential synergies with systematic reviewing. The possibility of fruitful collaborations between evidence mappers and systematic reviewers has been identified by other evidence mapping authors [4,5]. Given the resource-intensity of all secondary research methodologies, the strategic advantages and efficiencies of such collaborations are clear.

Two levels of data extraction have been explored by the GEM Initiative; an overview of interventions and study design, and a more in-depth description of study characteristics. These highlight the many potential outputs and applications of evidence maps and the ability of the evidence mapping process to be tailored to specific needs. In highlighting the usefulness of evidence maps in scoping research in broad topic areas, it is acknowledged that this breadth of coverage comes at the cost of evidence synthesis and quality appraisal, and therefore evidence maps cannot be used to guide clinical practice. This limitation should be borne in mind when reporting on and disseminating evidence maps.

Evidence maps complement other forms of reviews for use by researchers, clinicians, patients, carers and policy-makers with an interest in accessing and using evidence resources.

## Appendix 1 - Examples of search and selection procedures used by the GEM Initiative

### Step 3A: Search Strategy: Intervention Search string for 'spasticity' used in Medline (April 2008)

1. Muscle Spasticity/

2. spastic*.mp.

3. 1 or 2

4. Botulinum Toxin Type A/

5. exp GABA Agonists/

6. exp gamma-Aminobutyric Acid/

7. "gamma amino butyric acid*".mp.

8. or/4-7

9. "Range of Motion, Articular"/

10. stretch*.mp.

11. Splints/

12. position*.mp.

13. exp Physical Therapy Modalities/

14. physiotherap*.mp.

15. Occupational Therapy/

16. or/9-15

17. Rhizotomy/or rhizotomy.mp.

18. myelotomy.mp.

19. Cordotomy/or cordotomy.mp.

20. cordectomy.mp.

21. or/17-20

22. or/8,16,21

23. 3 and 22

### Step 3B: Inclusion criterion: 'Rehabilitation' phase of care

Subacute care and long term care through community management. Subacute care is defined as medical and skilled nursing services provided to patients who are not in an acute phase of an illness but who require a level of care higher than that provided in a long term care setting. Long term care includes community management and is defined as extended care for chronic conditions or disabilities requiring periodic, intermittent, or continuous care [21].

### Step 3C: Databases searched for TBI rehabilitation question: What are effective interventions for preventing and managing skeletal muscle spasticity?

*Major medical databases*:

Cumulative Index to Nursing and Allied Health Literature (CINAHL), OVID Embase, OVID Medline, PsycINFO, Cochrane Library, ISI Web of Science, PubMed

*Non-English databases*:

India Med, Korea Med, LILACS, Panteleimon

*Trials registries*:

WHO - International Clinical Trials Registry Platform (ICTRP), The UK National Research Register

*Further databases (to reflect the multidisciplinary nature of this intervention)*:

Physiotherapy Evidence Database (PEDro), OTSeeker, PsycBITE

### Steps 3D - 3F: Search and selection results for TBI rehabilitation question: What are effective interventions for preventing and managing skeletal muscle spasticity?

673 titles/abstracts screened

152 full-text articles ordered and reviewed

61 articles included in evidence map (one ongoing trial and three non-English studies were also identified)

## Competing interests

The authors declare that they have no competing interests.

## Authors' contributions

RG and AC conceived the GEM Initiative; all authors were involved in undertaking research for the GEM Initiative and developing its evidence mapping methods; PB drafted the manuscript; all authors read and approved the final manuscript.

## Pre-publication history

The pre-publication history for this paper can be accessed here:

http://www.biomedcentral.com/1471-2288/11/92/prepub

## References

[B1] Hagell A Bourke Dowling S Scoping Review of Literature on the Health and Care of Mentally Disordered Offenders 1999 York: NHS Centre for Reviews and Dissemination

[B2] Peersman G A Descriptive Mapping of Health Promotion Studies in Young People EPPI Research Report 1996 London: EPI-Centre

[B3] Arksey H O'Malley L Scoping Studies: Towards a Methodological Framework International Journal of Social Research Methodology 2005 8 1 19 32 10.1080/1364557032000119616

[B4] Bates S Clapton J Coren E Systematic maps to support the evidence base in social care Evidence & Policy: A Journal of Research, Debate and Practice 2007 3 4 539 551 10.1332/174426407782516484

[B5] Katz DL Williams AL Girard C Goodman J Comerford B Behrman A Bracken MB The evidence base for complementary and alternative medicine: methods of Evidence Mapping with application to CAM Alternative therapies in health and medicine 2003 9 4 22 30 12868249

[B6] Collie A Gains in neurotrauma research activity and output associated with a Victorian state government funding program The Medical journal of Australia 2010 192 12 712 714 20565352 10.5694/j.1326-5377.2010.tb03709.x

[B7] Van de Ven A Delbecq A Nominal Versus Interacting Group Processes for Committee Decision-Making Effectiveness Academy of Management Journal 1971 June 203 212

[B8] Flemming K Asking answerable questions Evidence-Based Nursing 1998 1 2 36 37 10.1136/ebn.1.2.36

[B9] Straus S Richardson W Glasziou P Haynes R Evidence-Based Medicine: How to Practice and Teach EBM 2005 Edinburgh: Elsevier

[B10] World Health Organization Towards a Common Language for Functioning, Disability and Health: ICF The International Classification of Functioning, Disability and Health 2002 Geneva: World Health Organization

[B11] ICF Browser http://www.who.int/classifications/icfbrowser/

[B12] Arksey H Scoping the field: services for carers of people with mental health problems Health & social care in the community 2003 11 4 335 344 10.1046/j.1365-2524.2003.00433.x 14629205

[B13] Jones T Evans D Conducting a systematic review Australian Critical Care 2000 13 2 66 71 10.1016/S1036-7314(00)70624-2 11235454

[B14] The Cochrane Collaboration Cochrane handbook for systematic reviews of interventions 2008 Chichester, England: John Wiley & Sons

[B15] Parkhill A Hill K Identifying the effective evidence sources to use in developing Clinical Guidelines for Acute Stroke Management: lived experiences of the search specialist and project manager Health Information and Libraries Journal 2008 26 1 47 55 19245643 10.1111/j.1471-1842.2008.00784.x

[B16] Kastner M Straus SE McKibbon KA Goldsmith CH The capture-mark-recapture technique can be used as a stopping rule when searching in systematic reviews Journal of clinical epidemiology 2009 62 2 149 157 10.1016/j.jclinepi.2008.06.001 18722088

[B17] Coleman K Grimmer-Somers K Hillier S Merlin T Middleton P Salisbury J NHMRC additional levels of evidence and grade for recommendations for developers of guidelines: Stage 2 consultation 2008 NHMRC (National Health and Medical Research Council)

[B18] NHMRC/VNI Fellowships & Awards http://www.vni.com.au/capacitybuilding/cid/301/parent/0/pid/6/t/capacitybuilding/title/nhmrcvni-fellowships-awards

[B19] Phillips K Pitt V O'Connor D Gruen R Interventions for managing skeletal muscle spasticity following traumatic brain injury [Protocol] Cochrane Database of Systematic Reviews 2011 1 10.1002/14651858.CD008929.pub2 PMC6486165 29165784

[B20] Parkhill A Clavisi O Pattuwage L Bragge P Tavender E Gruen R Is the highly sensitive search worth the effort? [Poster presentation] 17th Cochrane Colloquium 11 - 14 October 2009, Singapore 2009

[B21] Khan F Baguley IJ Cameron ID 4: Rehabilitation after traumatic brain injury The Medical journal of Australia 2003 178 6 290 295 12633489 10.5694/j.1326-5377.2003.tb05199.x

[B22] Greenhalgh T Papers that summarise other papers (systematic reviews and meta-analyses) British Medical Journal 1997 315 7109 672 675 9310574 10.1136/bmj.315.7109.672 PMC2127461

[B23] Mortenson PA Eng JJ The use of casts in the management of joint mobility and hypertonia following brain injury in adults: A systematic review Phys Ther 2003 83 7 648 658 12837126

[B24] Ayas S Leblebici B Sozay S Bayramoglu M Niron EA The effect of abdominal massage on bowel function in patients with spinal cord injury Am J Phys Med Rehabil 2006 85 12 951 955 10.1097/01.phm.0000247649.00219.c0 17117000

[B25] Furusawa K Sugiyama H Ikeda A Tokuhiro A Koyoshi H Takahashi M Tajima F Autonomic dysreflexia during a bowel program in patients with cervical spinal cord injury Acta Med Okayama 2007 61 4 221 227 17726511 10.18926/AMO/32867

[B26] Luther SL Nelson AL Harrow JJ Chen F Goetz LL A comparison of patient outcomes and quality of life in persons with neurogenic bowel: standard bowel care program vs colostomy J Spinal Cord Med 2005 28 5 387 393 16869085 10.1080/10790268.2005.11753838 PMC1808270

